# Genetics of 21-OH Deficiency and Genotype–Phenotype Correlation: Experience of the Hellenic National Referral Center

**DOI:** 10.3390/cimb46100635

**Published:** 2024-09-24

**Authors:** Irene Fylaktou, Anny Mertzanian, Ioanna Farakla, Alexandros Gryparis, Ioannis Anargyros Vasilakis, Maria Binou, Evangelia Charmandari, Christina Kanaka-Gantenbein, Amalia Sertedaki

**Affiliations:** 1 Division of Endocrinology, Diabetes and Metabolism ‘Aghia Sophia’ Children’s Hospital ENDO-ERN Center for Rare Paediatric Endocrine Diseases, First Department of Pediatrics, Medical School, National and Kapodistrian University of Athens, ‘Aghia Sophia’ Children’s Hospital, 11527 Athens, Greece; annymertz@med.uoa.gr (A.M.); ioanna.farakla@gmail.com (I.F.); vasilakisioan@yahoo.gr (I.A.V.); mmpinou@hotmail.com (M.B.); evangelia.charmandari@googlemail.com (E.C.); ckanaka@med.uoa.gr (C.K.-G.); aserted@med.uoa.gr (A.S.); 2 Department of Speech and Language Therapy, School of Health Sciences, University of Ioannina, 45110 Ioannina, Greece; alexandros@uoi.gr

**Keywords:** *CYP21A2* gene, 21-hydroxylase deficiency, congenital adrenal hyperplasia, pathogenic variants, 17-hydroxyprogesterone, de novo aberrations

## Abstract

21-hydroxylase deficiency (21-OHD) represents the most common form of congenital adrenal hyperplasia (CAH) due to *CYP21A2* gene pathogenic variants. Τhe aim of this study was the identification of *CYP21A2* variants in 500 subjects of Greek origin with a suspicion of 21-OHD and, by using the existing hormonal assessment and genotypes of the 500 subjects tested, to identify a biomarker that could differentiate between the heterozygotes and the cases with no pathogenic variants identified. Five hundred subjects with clinical suspicion of 21-OHD underwent *CYP21A2* gene sequencing and Multiplex Ligation Dependent Probe Amplification (MLPA). Genetic diagnosis was achieved in 27.4% of the subjects tested, most of which presented with the non-classic form (NC) of 21-OHD. Heterozygotes accounted for 42.6% of cases, whereas no pathogenic variants were identified in 27% of cases. De novo aberrations, duplications, and five novel variants were also identified. Statistical analysis revealed that the difference between the basal and 60′ post-ACTH stimulation 17-hydroxyprogesterone concentrations (Δ17-OHP^60-0^) could be a potential biomarker (*p* < 0.05) distinguishing the heterozygotes from the cases with no pathogenic variants identified, although no clear cut-off value could be set. Further analysis revealed overlapping clinical manifestations among all the subjects tested. The presented phenotypic traits of the subjects tested and the inability to identify a discriminative biochemical marker highlight the importance of comprehensive *CYP21A2* genotyping to ascertain the correct genetic diagnosis and proper genetic counselling.

## 1. Introduction

Congenital adrenal hyperplasia (CAH) is a group of autosomal recessive disorders caused by pathogenic variants identified in seven genes (*CYP21A2*, *CYP11B1*, *CYP17A1*, *HSD3B2*, *StAR*, *POR*, and *CYP11A1*) involved in the steroidogenesis pathway [1]. Most CAH cases (95%) are the result of pathogenic variants in the *CYP21A2* gene encoding for the enzyme 21-hydroxylase [2,3].

The enzyme 21-hydroxylase is responsible for the conversion of 17-hydroxyprogesterone (17-OHP) and progesterone to 11-deoxycortisone and 11-deoxycorticosterone, respectively. Reduced 21-hydroxylase function leads to inadequate cortisol and aldosterone synthesis, resulting in the excessive accumulation of the precursors 17-OHP and progesterone thus directing steroidogenesis towards the uninhibited androgen production, and finally leading to the excess concentration of adrenal androgens, responsible for the clinical picture of affected individuals.

Depending on the degree of enzymatic activity and consequently on the clinical manifestations, CAH is classified in two forms, the non-classic (NC) and classic (C) forms.

The classic form, with an incidence of 1:14,000–1:18,000 live births, based on neonatal screening, is further divided into the classic salt wasting (SW) and the classic simple virilizing (SV) forms [4]. In classic 21-OHD (SW and SV), females present with atypical/ambiguous genitalia at birth due to the intrauterine severe virilization of the external genitalia because of the prenatal excess androgen production and action, while males present with an enlarged penis, that quite often escapes proper medical attention. The SW form is further characterized after birth with severe salt loss, failure to thrive, potentially fatal hypovolemia, and shock due to both cortisol and aldosterone insufficiency. Therefore, in both sexes, the SV form is mainly characterized by the signs of excess androgen production without salt loss since patients exhibit sufficient aldosterone production. The NC or late onset 21-OHD is considered to be the most frequently occurring endocrine disorder, with a wide range of incidence of 1:200 to 1:1000 in various ethnic groups (Caucasians, Askenaki Jews, Yugoslavs, Mediterranean, Hispanics and Anglo-Saxons) [5,6]. The NC form is manifested through a variety of clinical signs ranging from the absence of clinical signs to premature adrenarche, hirsutism, acne, advanced bone age, and menstrual irregularities [7].

However, the boundaries between the different forms of CAH are not strictly defined, and it is therefore advisable to consider CAH as a continuum of phenotypes from asymptomatic to mild and severe [8].

Confirmation of 21-OHD is based on the serum 17-OHP concentrations, both basal and after an ACTH stimulation test [9]. More precisely, basal 17-OHP concentrations of <2 ng/mL (6nM) have been proposed for the exclusion of 21-OHD, with a more accurate measurement for 17-OHP concentrations after the ACTH stimulation test to be >10 ng/mL (30nM) for the diagnosis of 21-OHD [9,10,11]. Although 17-OHP concentration is currently used for 21-OHD diagnosis, the effect of several factors on 17-OHP limits its diagnostic utility [9]. To date, comprehensive *CYP21A2* genotyping remains the gold standard for confirming or excluding 21-OHD [12]. The *CYP21A2* gene is located on the short arm of chromosome 6 (6p21.31) in a genetic unit known as the RCCX module, whereas its highly homologous nonfunctional pseudogene, *CYP21A1P*, is located 30 kb upstream [13,14,15,16].

Most pathogenic variants (75%) in the *CYP21A2* gene derive from gene conversion events between the pseudogene and the *CYP21A2* gene, while 5–10% of cases represent novel or rare variants [17,18]. Furthermore, gene deletions, duplications, and *CYP21A1P–CYP21A2* chimeras have also been reported, while de novo aberrations account for 1–2% of the cases [18,19,20,21,22,23,24]. Classification of the *CYP21A2* gene pathogenic variants is based on their effect on the 21-hydroxylase enzymatic activity. Null Group variants lead to complete inactivation of the enzyme, whereas Group A variants reduce the enzymatic activity to less than 1%. Both groups are associated with the SW form of the disease. Group B variants reduce the enzymatic activity to 2–5% and are associated with the SV form of the disease, while Group C variants result in an enzymatic activity of 20–50% and are associated with the NC form of the disease [7]. On the contrary, *CYP21A2* gene duplications do not have any effect on the 21-hydroxylase enzymatic activity. It has been previously reported that *CYP21A2* gene duplications consist of a wild-type *CYP21A2* copy and a second *CYP21A2* copy carrying one or more pathogenic variants, with the p.Q319* pathogenic variant being more frequently presented [20,21,22,23,24]. *CYP21A2* gene duplication with the p.Q319* on the same allele do not affect the enzymatic activity of the 21-hydroxylase thus representing a normal functional allele [25].

Most affected individuals are compound heterozygotes and the patient’s phenotype is defined by the less severe pathogenic variant, with most cases presenting a good genotype–phenotype correlation. However, cases of genotype–phenotype discordance have been reported [26,27,28].

The aim of this study is to present the results of *CYP21A2* genotyping in 500 Greek subjects referred with the clinical suspicion of CAH. Furthermore, we aim to identify a biomarker that could differentiate between *CYP21A2* heterozygotes and cases with no pathogenic variants identified based on their clinical and hormonal profiles.

## 2. Materials and Methods

### 2.1. Subjects

A total of 539 individuals of Greek origin with suspicion of 21-OHD were referred to our center for comprehensive *CYP21A2* genotyping during the last 7 years (2017–2023) after clinical and hormonal assessments by their pediatrician/endocrinologist. Clinical assessment was based on CAH-indicative clinical features according to age of presentation as follows: During the neonatal period, the cardinal clinical features were salt-wasting and adrenal crisis, as well as atypical/ambiguous genitalia in females or penile enlargement in males; during childhood and adolescence, premature adrenarche, bone age advancement, hirsutism, acne, and menstrual irregularities were observed; and during adulthood, hirsutism, acne, menstrual irregularities or even subfertility would occur. Hormonal assessments included the basal 17-OHP, cortisol (F), Δ4-Androstenedione (Δ4-A), testosterone (T), dehydroepiandrosterone sulfate (DHEA-S), and adrenocorticotropic hormone (ACTH) levels, while most cases also underwent an ACTH stimulation test.

Out of the 539 subjects referred, 39 were excluded due to insufficient clinical/hormonal data. Those 39 cases were either referred to our center for the exclusion of 21-OHD to confirm the diagnosis of Polycystic Ovary Syndrome (PCOS) or excluded due to the absence of hormonal data (basal 17-OHP, F, Δ4-A, T, DHEA-S) or due to the absence of CAH-indicative clinical features. The remaining 500 subjects were categorized into three groups according to their age at the time of the referral/clinical diagnosis. Age Group I included patients referred during infancy (n = 32/500), Age Group II included subjects referred during childhood/early adolescence (1–13 years, n = 405/500) while Age Group III consisted of subjects in late adolescence (13–18 years, n = 63/500).

The median age of the 500 cases (378 females, 122 males) was 8.5 years (range: 3 days-18 years). A total number of 926 parents were also analyzed.

The study protocol has been approved by the ethical committee of the “Aghia Sophia” Children’s Hospital. Written informed consent was obtained from all participants and/or their legal guardians for the molecular analysis of the *CYP21A2* gene.

### 2.2. DNA Extraction

Genomic DNA was isolated from the peripheral blood samples, employing either the Maxwell^®^ 16 Blood DNA Purification Kit (Promega, Madison, WI, USA) or the QIAamp DNA Blood Mini Kit (Qiagen, Hilden, Germany) according to the manufacturer’s instructions.

### 2.3. CYP21A2 Gene Sequencing

PCR and bidirectional sequencing of the exons and introns of *CYP21A2* gene was carried out as previously described [10]. Purification of the PCR products was performed with the combination of two different enzymes [Exonuclease I/Shrimp Alkaline Phosphatase (rSAP), New England BioLabs, Ipswich, MA, USA].

Sequencing of the *CYP21A2* gene was carried out for all cases and their parents (when available). Analysis of the *CYP21A2* gene was based on the reference gene NG_007941.2 and transcript NM_000500.9.

In cases where two or more pathogenic variants were identified on the same allele, these were reported as multiple pathogenic variants and their impact on the enzymatic activity of the 21-hydroxylase was defined by the most severe variant. *CYP21A1P–CYP21A2* chimeras were also included in this category.

Negatively genotyped cases in the *CYP21A2* gene were categorized as cases with no pathogenic variants identified.

### 2.4. MLPA

Multiplex Ligation Dependent Probe Amplification (MLPA) employing the P050-C1 CAH kit (MRC Holland, Amsterdam, The Netherlands) was carried out in cases with a suspicion of duplication/deletion of the *CYP21A2* gene after sequencing analysis was performed.

The cases in which duplication of the gene (with or without the p.Q319* on the same allele) was observed either in heterozygosity or *in trans* with a pathogenic variant, were not considered as cases with no pathogenic variants identified or as solely heterozygotes but formed a distinct group, referred to as cases with duplication of the *CYP21A2 *gene.

### 2.5. Evaluation of Novel Variants

Novel variants were evaluated according to the ACMG guidelines [29]. The frequency of novel variants was also searched in the Genome Aggregation Database [30].

### 2.6. Statistical Analysis

Subjects carrying novel variants and duplication of the *CYP21A2* gene were excluded from the statistical analysis.

Statistical analysis for the 500 cases was performed using descriptive and inferential statistics. Descriptive statistics (including mean, median, SD, minimum, maximum, and IQR) were employed for the available clinical data of all the genotyped cases. Comparisons of the hormonal concentrations [basal 17-OHP, cortisol, Δ4-Androstenedione, DHEA-S, testosterone, and ACTH, as well as the difference between 60′ and basal 17-OHP concentrations after the ACTH stimulation test (Δ17-OHP^60-0^), and between 60′ and basal cortisol concentrations after the ACTH stimulation test (ΔF^60-0^)] amongst each of the three different subgroups of heterozygotes (comprising heterozygotes carrying pathogenic variants that are associated with the SW, SV, and NC forms, respectively) and the group of the cases with no pathogenic variants identified was performed using the Mann–Whitney U test (inferential statistics). The comparison was performed in groups of the same sex. Statistical analysis was performed using IBM SPSS v.28 (IBM Corp. Released 2021. IBM SPSS Statistics for Windows, Version 28.0. Armonk, NY, USA: IBM Corp) and RStudio version 2023.06.0 Build 421 [RStudio Team (2020). RStudio: Integrated Development for R. RStudio, PBC, Boston, MA, USA URL http://www.rstudio.com/ (accessed on 15 July 2023)]. *p*-values < 0.05 were considered statistically significant.

## 3. Results

**Figure 1:**
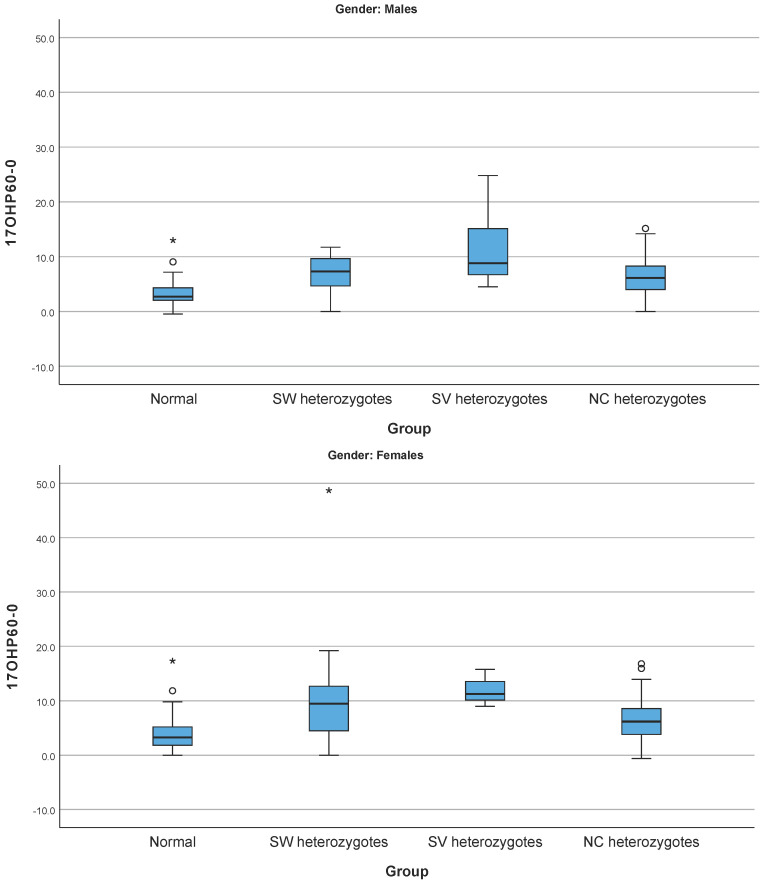
Box plots of Δ17-OHP^60-0^ in the group of normal (cases with no pathogenic variants identified) and the three groups of heterozygotes for pathogenic variants related to SW, SV, and NC forms of 21-OHD in males and females. Cycles represent extreme values while asterisks represent very extreme values. Normal indicates the cases with no pathogenic variants identified. The horizontal black lines in every group represent the corresponding median values. As shown, although there is a statistically significant differences between the median values among the groups, there is an overlap in the range of the Δ17-OHP^60-0^ values in the cases with no pathogenic variants identified with each group of the heterozygotes.

The statistical analysis performed for the 500 cases studied using the different hormonal concentrations (17-OHP, Δ17-OHP^60-0^, F, ΔF^60-0^, Δ4-A, DHEA-S, testosterone, and ACTH) amongst cases with no pathogenic variants identified and the different subgroups of heterozygotes revealed that the Δ17-OHP^60-0^ exhibited a statistically significant difference (*p* < 0.05) in both sexes as depicted in Figure 1. However, the great overlap in the Δ17-OHP^60-0^ values amongst all the subgroups of heterozygotes when compared to the group of cases with no identified pathogenic variants in both sexes did not allow us to set discriminating cut-off values.

**Figure 2:**
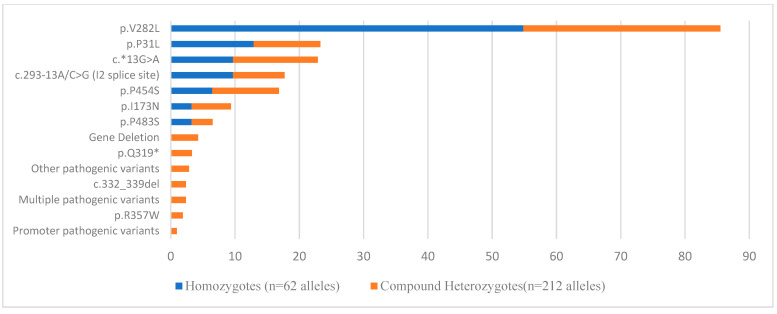
Frequency of *CYP21A2* gene pathogenic variants identified in 21-OHD patients. The blue label represents the homozygotes, and the orange label represents the compound heterozygotes.

Genetic diagnosis of 21-OHD was achieved in 137 subjects (27.4%), who were genotypically characterized either as compound heterozygotes (n = 106, 21.2%) or homozygotes (n = 31, 6.2%) (Figure 2). Heterozygotes accounted for 42.6% (213/500) of the tested population, while in 27% (135/500), no pathogenic variant was identified. Duplication of the *CYP21A2* gene (with or without the p.Q319* in the same allele) was observed in 2.6% (13/500) of the cases.

The classic (SW or SV) form of 21-OHD was diagnosed in 10.2% of cases (14/137)—9.4% (10/106) of the compound heterozygotes and 12.9% (4/31) of the homozygotes. The majority of SW and SV cases carried the c.293-13C>G (I_2_splice site) pathogenic variant (Group A) and the p.I173N pathogenic variant (Group B) (Table A1) respectively. Most classic 21-OHD patients belonged to Age Group I (64.3%, 9/14).

**Figure 3:**
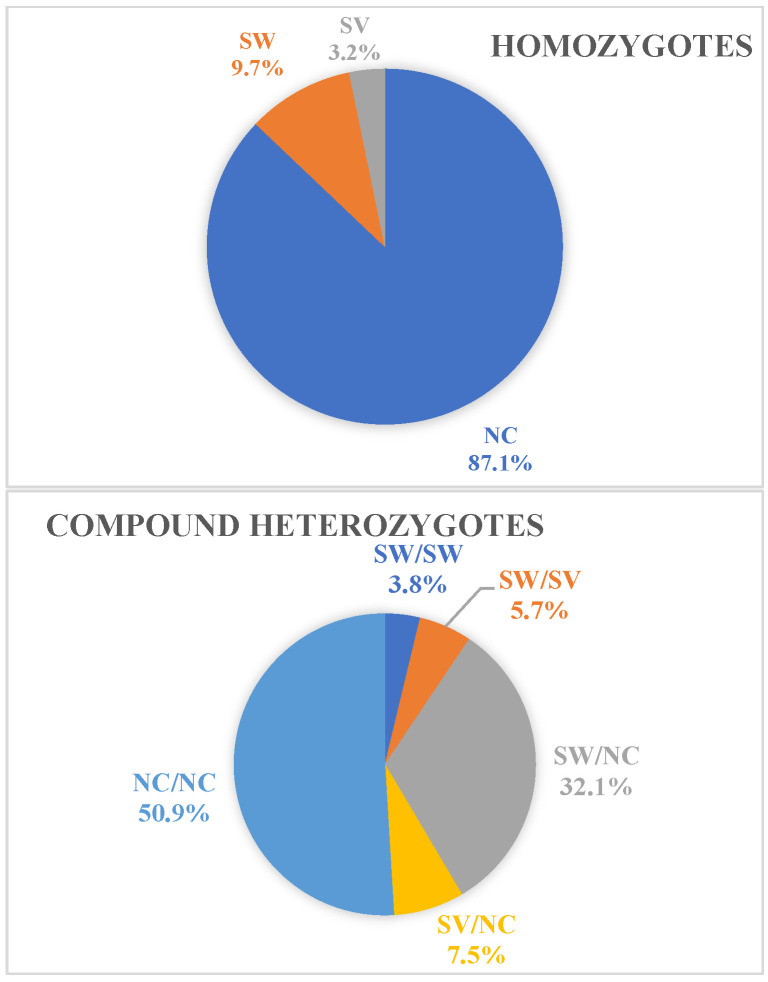
The distribution of 21-OHD forms in homozygotes and compound heterozygotes.

**Figure 4:**
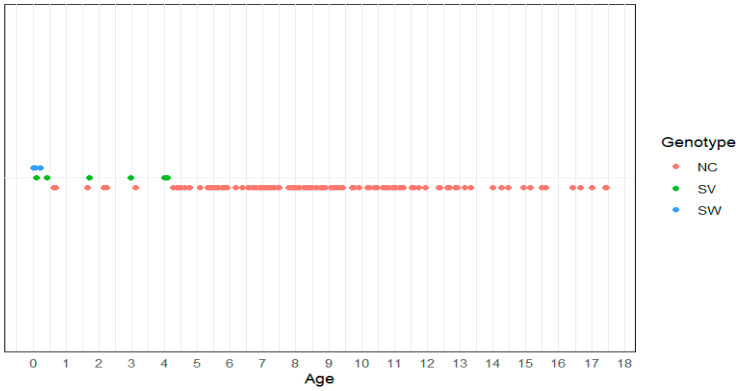
Patients’ age (years) at diagnosis in the different forms of 21-OHD.

The NC form was observed in 89% of patients—90.6% (96/106) of the compound heterozygotes and 87.1% (27/31) of the homozygotes (Figure 3). Group C pathogenic variants were identified in 59.2% (81/137) of patients; 24.8% (34/137) harbored a Group C *in trans* with a Group null/A pathogenic variant and 5.9% (8/137) harbored a Group C *in trans* with a Group B pathogenic variant. Most cases presenting with the NC form of 21-OHD belonged to Age Groups II and III, with a frequency of 77.3% (106/137) and 11% (15/137), respectively. The age distribution of all patients is presented in Figure 4.

**Figure A1:**
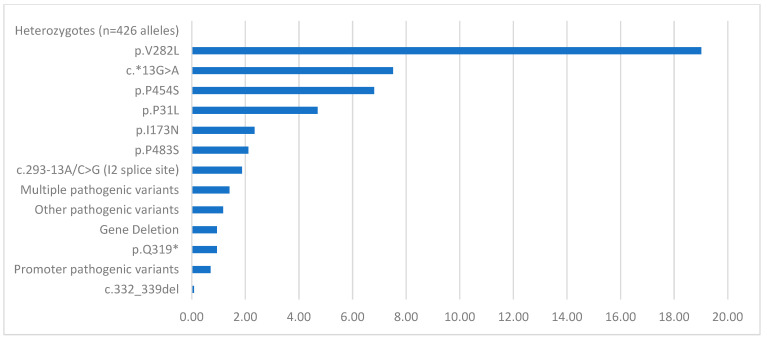
Frequency of *CYP21A2* gene pathogenic variants in heterozygotes and cases with duplication of the *CYP21A2* gene.

The most common pathogenic variant identified in patients, heterozygotes, and cases with *CYP21A2* gene duplication was the p.V282L (Group C variant) with an allele frequency of 33.7% and 19%, respectively (Figure 2 and Figure A1). Most heterozygotes (82.3%, 190/213) belonged to Age Group II.

**Figure 5:**
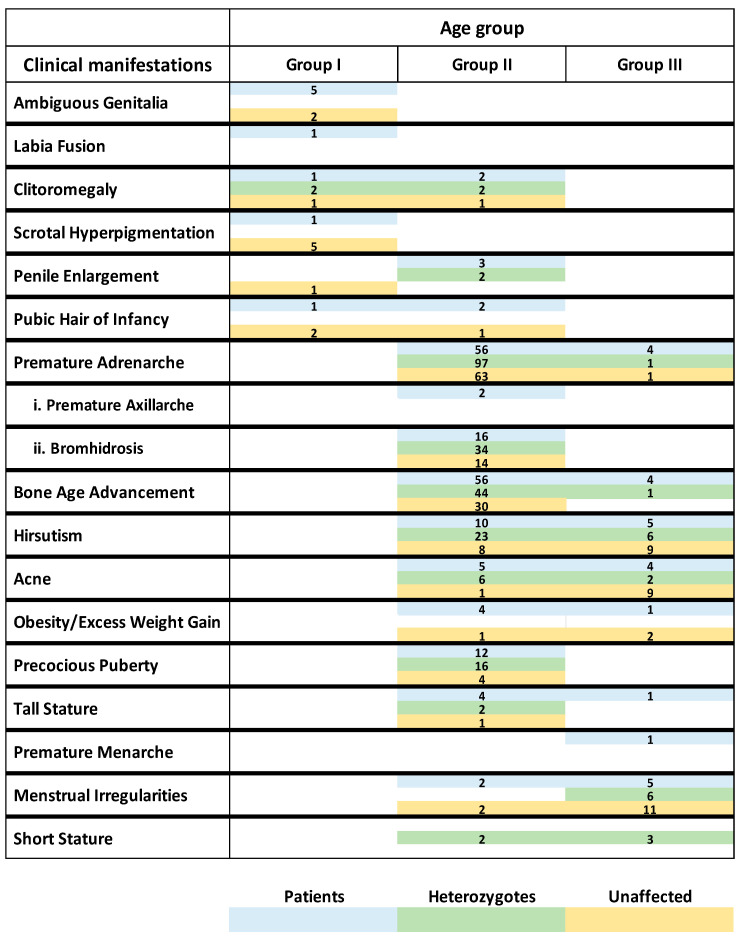
Clinical manifestations of 21-OHD patients, heterozygotes and cases with no pathogenic variants identified (unaffected) distributed according to their age group.

The hormonal profile and clinical manifestations categorized according to age group (Age Group I, Age Group II, and Age Group III) and genotypic group (patients, heterozygotes, and cases with no pathogenic variants identified) are depicted in Table 1, Table 2 and Table 3 and Figure 5.

Moreover, in the study, five novel variants were identified, most located in the gene’s promoter region (Table 4). Two of these novel variants were found *in trans* with a known pathogenic variant, while the other three were found in heterozygosity (Table 4). The GnomAD frequency of the novel variants (apart from the c.-82C>T) was low or not reported (Table 4).

De novo pathogenic variants/rearrangements were identified in four cases (one compound heterozygote and three heterozygotes), accounting for 0.4% of the alleles tested (4/1000 alleles). The compound heterozygote was found to harbor the maternal p.V282L and a de novo deletion of the *CYP21A2* gene whereas the father was carrying the Q319* *in cis* with duplication of the gene. Among the three heterozygotes, one case harbored a de novo deletion of the *CYP21A2* gene, while the other two cases carried the de novo p.I173N and c.293-13C>G pathogenic variants, respectively. The analysis of the heterozygotes’ parents revealed a duplication of the *CYP21A2* gene in the father of the heterozygote carrying the de novo deletion of the *CYP21A2* gene.

Duplications were observed in 2.6% (13/500) of the cases with 0.8% (4/500) carrying a duplication of the *CYP21A2* gene with the p.Q319* *in trans* with a known pathogenic variant. It is worth mentioning that in one case, a duplication of the gene was observed along with multiple pathogenic variants including the p.Q319* on the same copy of the gene.

In the present study, genetic analysis of the entire *CYP21A2* gene was performed in 926 parents, with 2.7% (25/926) of them depicted to be homozygotes/compound heterozygotes of pathogenic, VUS, or novel variants. More precisely, 2.5% (23/926) of the parents were found to harbor pathogenic variants resulting in the NC form (homozygotes/compound heterozygotes), identified solely by genotyping (without any clinical manifestation reported); 0.7% (6/926) harbored a pathogenic variant related to the classical form *in trans* with a variant related to the NC form; and 0.2% (2/926) carried novel variants or VUS *in trans* with a known pathogenic variant.

## 4. Discussion

In the current study, the genetic analysis of 500 subjects with the clinical suspicion of CAH is presented. Patients accounted for 27.4% of cases studied, with most of them presenting with the NC form of 21-OHD, while both the SW and SV forms of 21-OHD were underrepresented. The categorization of 21-OHD patients in different age groups confirmed that the severity of clinical manifestations due to the reduced 21-OH activity and the diagnosis of 21-OHD is directly related to the age at presentation. As expected, the more severe classical form will be diagnosed earlier in life, while the NC form will be diagnosed later, revealing the continuum of 21-OHD through all age groups.

In the current study, different adrenal hormones were assessed to identify a biomarker that could distinguish the heterozygotes of 21-OHD from the cases harboring no pathogenic variants. The Δ17-OHP^60-0^ in both sexes was found to be a potential biomarker; however, the overlap in its values between the different groups did not allow us to set cut-offs to discriminate them. This overlap in the values of Δ17-OHP^60-0^ of the group of cases with no pathogenic variants identified and the different groups of heterozygotes shows that this biomarker is not suitable to differentiate heterozygotes from the cases with no pathogenic variants identified, thus it cannot be used in clinical practice. To date, several studies have attempted to distinguish heterozygotes from the cases harboring no pathogenic variants using 17-OHP concentrations as a biomarker with no clear results. This could be attributed to several limitations such as the assay used (RIA) for 17-OHP measurements, the variability of the measurements amongst laboratories, the factors affecting the 17-OHP concentrations, as well as the 17-OHP as a biomarker itself. In this study, high 17-OHP concentrations were observed both in heterozygotes (mean 17-OHP concentrations in Age Group I: 11 ng/mL, Age Group II: 3.1 ng/mL, and Age Group III: 3.8 ng/mL) and cases with no pathogenic variants identified (mean 17-OHP in Age Group I: 6.8ng/mL, Age Group II: 2.7ng/mL, and Age Group III: 4.9 ng/mL), rendering the use of 17-OHP concentrations a non-reliable biomarker and confirming comprehensive *CYP21A2* genotyping as the gold standard for their distinction.

As previously mentioned, *CYP21A2* genotyping revealed a high percentage of the heterozygotes (42.6%) and the cases without any pathogenic variant (27%), although their hormonal profile and clinical features were indicative of 21-OHD. In this study, the clinical manifestations (ranging from severe to mild) of compound heterozygotes/homozygotes, heterozygotes, and cases with no pathogenic variants identified overlapped in each age group, particularly in Age Group II (comprising 81.2% of all cases).

Specifically, severe clinical manifestations, such as clitoromegaly or atypical/ambiguous genitalia, present in Age Groups I and II were found not only in the compound heterozygotes/homozygotes but also in the heterozygotes and the cases harboring no pathogenic variants. Likewise, the mild clinical manifestations (Age Group II and III) such as premature adrenarche were also present in all genotypic groups. In heterozygotes, the clinical features could be explained by the dominant-negative effect of certain pathogenic variants (such as the p.V282L), which affects the function of the wild-type allele thus reducing the enzymatic activity of 21-hydroxylase [33,34].

The number of cases without any identifiable pathogenic variants reported in this study is not negligible, and this could be explained by the fact that the CAH-related clinical features may not be exclusively indicative of 21-OHD. One of the main clinical manifestations observed in Age Group II and III was premature adrenarche that can also be the result of several other factors such as childhood obesity or being born IUGR (Intrauterine Growth restricted).

Moreover, pathogenic variants in other CAH genes involved in the steroidogenic pathway, the presence of other clinical entities (such as polycystic ovarian syndrome), epigenetic influences as well as environmental factors might also account for the phenotypic characteristics indicative of CAH.

The observed overlap in the clinical manifestations of all the genotypic groups and the inability to fully differentiate the heterozygotes from the cases without any pathogenic variants identified indicate that a more specific scoring system should be established to reduce the need for comprehensive *CYP21A2* genotyping.

To date, more than 200 pathogenic variants have been identified in the *CYP21A2* gene, with no hot spot regions reported [12,19,35]. The most common pathogenic variant found in our cohort to be related to the NC form of 21-OHD was the p.V282L, which is in concordance with the previous studies in the Greek population [10,36,37]. The frequency of the p.V282L in the Greek population (overall frequency: 24.4% in patients and heterozygotes) is similar to the Italian (23.9%) population but lower than the Cypriot (43.7–60%), Spanish (47.3%), and Portuguese (41.3%) populations [38,39,40,41,42].

The most common pathogenic variant identified in our cohort related to the SW form was the c.293-13C>G [I_2_splice site] (7.3%) with an incidence that is lower than in the Spanish (12.3%), the Portuguese (14.1%), the Turkish (22–33.2%), and the Croatian (35.5%) population [38,41,43,44,45,46].

Genotyping the *CYP21A2* gene led to the identification of five novel variants. Three of them located in the promoter region and which are crucial for the transcriptional activity of the gene, the c.-127G>A and c.-82C>T in heterozygosity and the c.-115G>T in compound heterozygosity with the pathogenic variant p.P31L, were classified as VUS according to the ACMG criteria due to insufficient or conflicting evidence. The other two novel variants were located in exon 7; the p.R255K in heterozygosity was classified as VUS and the p.V282M in compound heterozygosity with p.L308Ffs*6 was classified as Likely Pathogenic. Considering the hormonal data of the patients carrying these novel variants (presented in Table 4), only the p.V282M variant certainly contributes to the patient’s phenotype, and this patient was clinically considered and treated as the non-classic form. It should be noted that the V282 residue is located in a very restricted space at the I-helix, and we can assume that the substitution of Valine by Methionine could have the same effect as the substitution of Valine by Leucine due to the increased chain length of both the Methionine and Leucine when compared to Valine [47]. The patient carrying the c.-115G>T in compound heterozygosity with the p.P31L, presented increased 17-OHP concentrations with normal cortisol values and was therefore considered as heterozygote, since there was no evidence for the effect of the VUS variant. The three remaining VUS variants were only present in heterozygosity and therefore, irrespective of their potential impact on the enzymatic activity, their clinical significance is negligible.

Overall, in vitro studies are required to elucidate the pathogenicity of these variants and to clarify their impact on the enzymatic activity of 21-hydroxylase.

Different copy number variations at the RCCX model have been described due to unequal meiotic crossing-over events. Duplications of the *CYP21A2* gene along with the p.Q319* (trimodular haplotype) are known to be present in less than 2% of the general population except for the Spanish population [24,48]. In this study, duplications of the *CYP21A2* gene (with or without the p.Q319*) were found in a slightly higher frequency, emphasizing the importance of comprehensive *CYP21A2* genotyping, particularly in cases where the p.Q319* is present in order to provide proper genetic diagnosis and counselling [49]. The identification of duplications is also important for family planning since duplications are known to predispose to de novo aberrations in the offspring [50]. De novo aberrations are considered to occur due to unequal crossing over at the RCCX unit [17,18,51,52]. In this study, de novo aberrations were identified in 0.8% of subjects tested. We could speculate that these de novo aberrations probably occurred in the paternal gametic line since the fathers of the subjects tested to be carrying de novo deletion harbored duplications of the *CYP21A2* gene. To date, de novo aberrations have only been reported in maternal alleles carrying duplications of the *CYP21A2* gene; however, our findings indicate that de novo aberrations might also be the result of unequal crossing over events in the paternal alleles [50].

The identification of parents that harbor pathogenic variants in homozygosity/compound heterozygosity resulting in the NC form of 21-OHD, without any clinical manifestations, has also been previously reported [53,54]. This finding should be strongly considered when parents are only tested for the specific pathogenic variants identified in their offspring.

## 5. Conclusions

In conclusion, to the best of our knowledge, this is the largest study of the *CYP21A2* gene conducted to date in Greece revealing that, in our population, 21-OHD is mainly presented with the NC form. Despite the small sample size of SW and SV patients, the continuum of clinical manifestations in the 21-OHD patients is evident among the different age groups. The identification of four cases with de novo aberrations and parents with the NC form (without reported clinical manifestations), the slightly higher frequency of duplications in the *CYP21A2* gene, the high percentage of heterozygotes and cases without any pathogenic variants identified, as well as the inability to identify a discriminative biochemical marker to distinguish the different groups, underline the necessity for comprehensive *CYP21A2* genotyping of the family (trios) for proper diagnosis and genetic counselling.

## Figures and Tables

**Table 1:** Hormonal data presented among patients, heterozygotes, and cases with no pathogenic variants identified in Age Group I.

	Age Group I Patients			Age Group I Heterozygotes			Age Group I Cases with No Pathogenic Variants Identified		
	Total	Males	Females	Total	Males	Females	Total	Males	Females
**17-OHP concentrations(ng/mL)** Valid % Median [min–max] IQR Mean ± SD	n = 7 63.6 35.9 [0.8–1.793] 64.9 287.7 ± 664.9	n = 1 25 23 [23–23] 0 23	n = 6 85.7 38.1 [0.8–1.793] 85.5 331.8 ± 717	n = 2 50 11 [4.3–17.6] 6.6 11 ± 9.4	n = 0	n = 2 66.7 11 [4.3–17.6] 6.6 11 ± 9.4	n = 13 100 3.8 [1.7–19] 8.2 6.8 ± 5.7	n = 9 100 4.8 [1.7–19] 8.5 8.1 ± 6.4	n = 4 100 3.7 [3.3–4.3] 0.4 3.8 ± 0.4
**F concentrations (μg/dL)** Valid % Median [min–max] IQR Mean ± SD	n = 3 27.3 5.4 [3.3–19.4] 8.1 9.4 ± 8.8	n = 0	n = 3 42.9 5.4 [3.3–19.4] 8.1 9.4 ± 8.8	n = 3 75 15.9 [14.6–18.9] 2.2 16.5 ± 2.2	n = 0	n = 3 100 15.9 [14.6–18.9] 2.2 16.5 ± 2.2	n = 13 100 8 [0.9–23.1] 10.2 9.4 ± 7.3	n = 9 100 6.5 [0.9–20.2] 7.9 8.5 ± 6.8	n = 4 100 10.6 [1.7–23.1] 9.3 11.5 ± 9
**Δ4-A (ng/mL)** Valid % Median [min–max] IQR Mean ± SD	n = 4 36.4 6 [0.4–12.5] 5.6 6.2 ± 5.1	n = 0	n = 4 57.1 6 [0.4–12.5] 5.6 6.2 ± 5.1	n = 2 50 2.6 [0.1–5.2] 2.5 2.6 ± 3.6	n = 0	n = 2 66.7 2.6 [0.1–5.2] 2.5 2.6 ± 3.6	n = 8 61.5 0.4 [0–2.9] 2 1 ± 1.2	n = 4 44.4 0.2 [0–2.8] 0.9 0.8 ± 1.4	n = 4 100 1.1 [0.1–2.9] 1.6 1.3 ± 1.3
**Τ (ng/dL)** Valid % Median [min–max] IQR Mean ± SD	n = 3 27.3 36.5 [0.1–500] 249.9 178.9 ± 278.7	n = 0	n = 3 42.9 36.5 [0.1–500] 249.9 178.9 ± 278.7	n = 1 25 1.1 [1.1–1.1] 0 1.1	n = 0	n = 1 33.3 1.1 [1.1–1.1] 0 1.1	n = 10 76.9 62 [1.1–193] 74.4 69.2 ± 62.3	n = 6 66.7 68 [1.1–193] 108.6 78.8 ± 76.3	n = 4 100 61.5 [2.5–94] 31.1 54.9 ± 38.4
**ACTH (pg/mL)** Valid % Median [min–max] IQR Mean ± SD	n = 4 36.4 34.5 [2.5–163] 65.2 58.6 ± 72.5	n = 0	n = 4 57.1 34.5 [2.5–163] 65.2 58.6 ± 72.5	n = 1 25 130.9 [130.9–130.9] 0 130.9	n = 0	n = 1 33.3 130.9 [130.9–130.9] 0 130.9	n = 8 61.5 53.1 [9.9–91.4] 42.3 49.8 ± 29.8	n = 4 44.4 60.6 [17–91.4] 21.8 57.4 ± 30.7	n = 4 100 38 [9.9–82.8] 32.9 42.1 ± 31.2
**DHEAS (μg/dL)** Valid % Median [min–max] IQR Mean ± SD	n = 4 36.4 809 [2–2208.6] 1588.2 957.2 ± 1076.6	n = 0	n = 4 57.1 809 [2–2208.6] 1588.2 957.2 ± 1076.6	n = 2 50 61 [4.1–118] 57.0 61 ± 80.6	n = 0	n = 2 66.7 61 [4.1–118] 57 61 ± 80.6	n = 9 69.2 24 [0.2–2458.5] 978.5 563.4 ± 894.9	n = 6 66.7 5.2 [0.2–980] 74.1 181.4 ± 393.1	n = 3 75 1500 [24–2458.5] 1.217.2 1327.5 ± 1226.4

In patients, in Age Group I there were two female patients that exhibited values that could not be precisely determined by the methodology applied. More precisely, there was one female patient with DHEAS >1500 μg/dL that was set at 1500 μg/dL and one female patient that had Δ4-A > 12.5 ng/mL and was set at 12.5 ng/mL in order to proceed with the statistical analysis. Respectively, in cases with no pathogenic variants identified, there were two females and one male that exhibited higher or lower values. More precisely, in one female, the DHEAS was >1500 μg/dL and was set at 1500 μg/dL, the other female had Δ4-A < 0.1 ng/mL and T < 2.5 ng/dL and was set at 0.1 ng/mL and 2.5 ng/dL, respectively, and the male had Δ4-A < 0.006 ng/mL and was set at 0 ng/mL.

**Table 2:** Hormonal data presented among patients, heterozygotes, and cases with no pathogenic variants identified cases in Age Group II.

	Age Group II Patients			Age Group II Heterozygotes			Age Group II Cases with No Pathogenic Variants Identified		
	Total	Males	Females	Total	Males	Females	Total	Males	Females
**17-OHP concentrations (ng/mL)** Valid % Median [min–max] IQR Mean ± SD	n = 100 90.9 11 [0.8–200] 18.8 25.7 ± 40.1	n = 30 88.2 13.6 [1.9–200] 39.6 37.7 ± 52.3	n = 70 92.1 9.6 [0.8–200] 18.2 20.5 ± 32.7	n = 183 98.9 2.6 [0.3–13.7] 2.4 3.1 ± 2.1	n = 47 97.9 2.1 [0.4–7.5] 1.6 2.5 ± 1.5	n = 136 99.3 2.7 [0.3–13.7] 2.6 3.4 ± 2.3	n = 94 97.9 2.1 [0.5–25] 1.7 2.7 ± 2.8	n = 10 100 1.5 [0.5–25] 2.7 4.2 ± 7.5	n = 84 97.7 2.1 [0.5–8.9] 1.6 2.5 ± 1.5
**F concentrations (μg/dL)** Valid % Median [min–max] IQR Mean ± SD	n = 91 82.7 12.3 [4.2–26.5] 6.7 12.9 ± 4.9	n = 30 88.2 12.2 [4.2–91.5] 6.7 14.8 ± 15.5	n = 61 80.3 12.5 [5.2–26.5] 6.7 13.3 ± 4.6	n = 170 91.9 13.8 [3.9–29.5] 7.7 14.5 ± 5.6	n = 43 89.6 14 [5.3–23.8] 8.3 13.8 ± 5.3	n = 127 92.7 13.7 [3.9–29.5] 7.6 14.8 ± 5.6	n = 90 93.8 14.8 [0.6–31.5] 9.8 15.5 ± 6.5	n = 10 100 16.6 [2.9–26.7] 10.1 15.7 ± 7.9	n = 80 93 14.8 [0.6–31.5] 9.5 15.5 ± 6.4
**Δ4-A (ng/mL)** Valid % Median [min–max] IQR Mean ± SD	n = 71 64.5 0.8 [0–35.3] 1.4 1.7 ± 4.3	n = 21 61.8 0.9 [0.1–35.3] 1.7 3.1 ± 7.6	n = 50 65.8 0.8 [0–6.7] 1.1 1.2 ± 1.3	n = 117 63.2 0.4 [0–76] 0.6 1.2 ± 7	n = 29 60.4 0.4 [0–1.4] 0.6 0.5 ± 0.4	n = 88 64.2 0.5 [0–76] 0.6 1.5 ± 8.1	n = 73 76 0.4 [0.1–22] 0.5 0.9 ± 2.6	n = 7 70 0.3 [0.1–0.7] 0.3 0.4 ± 0.2	n = 66 76.7 0.4 [0.1–22] 0.6 1 ± 2.7
**Τ (ng/dL)** Valid % Median [min–max] IQR Mean ± SD	n = 88 80 8 [0–309] 16.5 20.6 ± 49.1	n = 31 91.2 3 [0–309] 22.6 36.0 ± 79.0	n = 57 75 8 [0–78.9] 15 12.3 ± 14.3	n = 144 77.8 5 [0–126] 12 10.6 ± 18.2	n = 41 85.4 7 [0–126] 16 18.2 ± 30.1	n = 103 75.2 3 [0–47] 11 7.6 ± 8.7	n = 83 86.5 2.5 [0–250] 5 9.5 ± 29.1	n = 10 100 2 [0–19] 2.2 3.5 ± 5.6	n = 73 84.9 2.5 [0–250] 5 10.4 ± 30.9
**ACTH (pg/mL)** Valid % Median [min–max] IQR Mean ± SD	n = 51 46.4 38.9 [2.3–357] 64.8 73.7 ± 80.6	n = 16 47.1 45.6 [2.3–357] 109.4 101 ± 104.8	n = 35 46.1 36.5 [10–310] 36.5 61.2 ± 64.7	n = 80 43.2 28.2 [0.6–400] 24.8 42.2 ± 49.8	n = 18 37.5 27.9 [8.8–120] 9.5 32.9 ± 24.7	n = 62 45.3 29.4 [0.6–400] 30 44.9 ± 54.9	n = 55 57.3 33 [0.7–229] 35.3 46.8 ± 44.3	n = 8 80 34.5 [13.8–153] 39.4 53.3 ± 46.4	n = 47 54.7 33 [0.7–229] 30.8 45.7 ± 44.4
**DHEAS (μg/dL)** Valid % Median [min–max] IQR Mean ± SD	n = 94 85.5 79.9 [0.1–3,742] 118.1 142.1 ± 390.1	n = 30 88.2 83.8 [0.4–448] 195.2 130.4 ± 135.6	n = 64 84.2 79.9 [0.1–3,742] 109.6 147.6 ± 464.9	n = 153 82.7 76.3 [0–964] 82 82.6 ± 93	n = 37 71.1 78 [1.2–252] 102 95.1 ± 68.9	n = 116 84.7 73.4 [0–964] 74.5 78.6 ± 99.4	n = 85 88.5 64.9 [0.3–341] 70.5 80.9 ± 60.8	n = 9 90 65 [0.5–195] 88.8 84.3 ± 63.6	n = 76 88.4 64.4 [0.3–341] 69.4 80.5 ± 60.9

In patients in Age Group II, 2 males were found to have concentrations that could not be precisely determined by the methodology applied. More precisely, there was one female and one male patient that exhibited concentrations of 17-OHP >200 ng/mL and the concentrations were set at 200 ng/mL. In heterozygotes, 5 females had concentrations that could not be precisely determined. Specifically, there were two females that were found to have T < 2.5 ng/dL and was set at 2.5 ng/dL, one female had T < 0.2 ng/dL and was set at 0.2 ng/dL, the fourth female had Δ4-A < 0.1 ng/mL and was set at 0.1 ng/mL, and the fifth female had Δ4-A < 0.3 ng/mL, T < 20 ng/dL and DHEAS < 15 μg/dL and were set at 0.3 ng/mL, 19 ng/dL, and 14 μg/dL, respectively. In cases with no pathogenic variants identified, 13 females and 3 males had concentrations that could not be precisely determined. Specifically, eight females and one male had T < 2.5 ng/dL and was set at 2.5 ng/dL, one female and one male had Δ4-A < 0.1 ng/mL and was set at 0.1 ng/mL, one male had T < 20 ng/dL and was set at 19 ng/dL, one female had Δ4-A < 0.3 ng/mL and T < 20 ng/dL and was set at 0.3 ng/mL and 19 ng/dL, respectively, two females had T < 0.025 ng/dL and was set at 0.02 ng/dL, and finally one female had Δ4-A < 0.1 ng/mL and T < 2.5 ng/dL and was set at 0.1 ng/mL and 2.5 ng/dL, respectively.

**Table 3:** Hormonal data presented among patients, heterozygotes, and cases with no pathogenic variants identified in Age Group III.

	Age Group III Patients			Age Group III Heterozygotes			Age Group III Cases with No Pathogenic Variants Identified		
	Total	Males	Females	Total	Males	Females	Total	Males	Females
**17-OHP concentrations (ng/mL)** Valid % Median [min–max] IQR Mean ± SD	n = 14 93.3 13.1 [1.7–119.5] 10.8 23.3 ± 31.8	n = 3 100 12 [1.7–20.6] 9.4 11.4 ± 9.4	n = 11 91.7 13.5 [3.4–119.5] 10 26.6 ± 35.2	n = 18 100 3.6 [0.8–7.8] 1.7 3.8 ± 1.7	n = 4 100 4.3 [3.3–5.2] 1.3 4.2 ± 0.9	n = 14 100 3.3 [0.8–7.8] 1.4 3.7 ± 1.9	n = 24 96 3.6 [0.6–20.9] 2.8 4.9 ± 4.5	n = 6 100 4.1 [2.6–13.1] 1.5 5.4 ± 3.9	n = 18 94.7 3.2 [0.6–20.9] 3 4.8 ± 4.8
**F concentrations (μg/dL)** Valid % Median [min–max] IQR Mean ± SD	n = 11 73.3 16.2 [4.6–27.9] 5.9 15 ± 6.4	n = 3 100 16.2 [6.5–17.3] 5.4 13.3 ± 5.9	n = 8 66.7 15.4 [4.6–27.9] 6 15.6 ± 6.8	n = 15 83.3 14.9 [9.8–23.2] 4.8 15.5 ± 4.3	n = 4 100 15.9 [9.8–23.2] 4.8 16.2 ± 5.5	n = 11 78.6 14.9 [10–23.1] 4.4 15.2 ± 4.1	n = 22 84.6 18.5 [5.4–29.5] 8.4 17.2 ± 6.1	n = 5 83.3 12.9 [11.1–18.6] 6.4 14.4 ± 3.6	n = 18 90 18.7 [5.4–29.5] 8.3 18 ± 6.4
**Δ4-A (ng/mL)** Valid % Median [min–max] IQR Mean ± SD	n = 9 60 3 [1.1–7.9] 1.7 3.4 ± 2	n = 3 100 1.3 [1.1–2.4] 0.6 1.6 ± 0.7	n = 6 50 3.9 [2.7–7.9] 1.1 4.3 ± 1.9	n = 10 55.6 2.3 [0.9–6.2] 1.6 2.8 ± 1.9	n = 2 50 2.1 [1.9–2.3] 0.2 2.1 ± 0.3	n = 8 57.1 2.6 [0.9–6.2] 2.6 3 ± 2.1	n = 19 76 2.4 [0.2–6.8] 2 2.8 ± 1.7	n = 4 66.7 0.7 [0.2–2.3] 0.6 1 ± 0.9	n = 15 78.9 2.6 [1.2–6.8] 2.4 3.3 ± 1.6
**Τ (ng/dL)** Valid % Median [min–max] IQR Mean ± SD	n = 13 86.7 18 [0.5–173] 70.3 47.3 ± 57.3	n = 3 100 133 [18–173] 77.5 108 ± 80.5	n = 10 83.3 15.5 [0.5–110] 38.8 29 ± 36.7	n = 16 88.9 30 [0.1–621] 55.5 126.6 ± 218.3	n = 4 100 531 [11.4–621] 228.9 423.6 ± 281.1	n = 12 85.7 26 [0.1–80] 38 27.6 ± 26	n = 18 72 26.5 [0.1–560.1] 42.6 78.7 ± 157.9	n = 3 50 452 [6.8–560.1] 276.6 339.6 ± 293.3	n = 15 78.9 25 [0.1–68] 36.2 26.5 ± 22.1
**ACTH (pg/mL)** Valid % Median [min–max] IQR Mean ± SD	n = 8 53.3 30.4 [15.4–142] 21.4 45.6 ± 40.8	n = 0	n = 8 66.7 30.4 [15.4–142] 21.4 45.6 ± 40.8	n = 10 55.6 28.6 [16.6–59.2] 14.9 32.3 ± 12.6	n = 1 25 31.6 [31.6–31.6] 0 31.6	n = 9 64.3 26 [16.6–59.2] 17.9 32.4 ± 13.3	n = 9 36 26.2 [10–84.8] 29.5 34.3 ± 24.6	n = 0	n = 9 47.4 26.2 [10–84.8] 29.5 34.3 ± 24.6
**DHEAS (μg/dL)** Valid % Median [min–max] IQR Mean ± SD	n = 13 86.7 243 [0.6–704] 275.6 280.6 ± 212.3	n = 3 100 243 [113.8–260] 73.1 205.6 ± 80	n = 10 83.3 246.5 [0.6–704] 274.6 303.1 ± 237.1	n = 16 88.9 209 [1.3–453] 164.5 189 ± 123.2	n = 3 75 255 [212–298] 43 255 ± 43	n = 13 92.9 131 [1.3–453] 159.5 173.8 ± 131.6	n = 22 88 196.5 [0.8–702] 247 227.9 ± 184.1	n = 4 66.7 139 [72–702] 219 263 ± 296.3	n = 18 94.7 229.5 [0.8–573.5] 241 220.1 ± 161.3

**Table 4:** Novel variants identified in the *CYP21A2* gene: clinical manifestations, hormonal profiles, and genotypes.

Variant	Location *CYP21A2*	Genotype	Clinical Manifestation	Sex	Hormonal Profile						Frequency (GnomAD)	ACMG Classification	Remarks
					Age	Hormonal Findings (before Treatment, if Required)	ACTH Stimulation Test						
								0′	30′	60′			
c.-127G>A	5’UTR	c.[-127G>A];[=]	PA	f	7 yrs	17-OHP: 4.98 ng/mL T: <0.025 ng/dL, DHEA-S: 61 μg/dL Δ4-A: 0.4 ng/mL	17-OHP (ng/mL)	4.91		11.88	-(not efficiently covered)	VUS (PM2, BP7)	Promoter region (c.-76 to c.-126)
							F (μg/dL)	18.3		26			
c.-115G>T	5’UTR	c.[-115G>T];[92C>T]	PA	f	6 yrs	-	17-OHP (ng/mL)	9.22		33.6	0.00003188	VUS (PM2, PM3, BP7)	Promoter region (c.-76 to c.-126)
							F(μg/dL)	12.99		23.43			
c.-82C>T	5’UTR	c.[-82C>T];[=]	PA	f	7 yrs	Δ4-A: 0.96 ng/mL, T: 6 ng/dL, DHEA-S: 178 μg/dL	17-OHP (ng/mL)	3.64	4.13	5.69	0.00223	VUS (BS1, BP7)	Promoter region (c.-76 to c.-126)
							F (μg/dL)	31.1	37.7	39.9			
c.764G>A# (p.R255K)	Exon 7	c.[764G>A];[=]	PHI	m	6 mos	17-OHP: 4 ng/mL, Cortisol: 14.7 μg/dL, ACTH: 99 pg/mL, PRA: 4.18 ng/mL/h, T: 0.88 ng/dL, DHEA-S: 246.8 μg/dL, Δ4-A: 0.76, Aldosterone: 71.5 ng/dL	17-OHP (ng/mL)	4.39		9.03	0.000004032	VUS (PM2)	[31] *
							F (μg/dL)	10.2		24.8			
c.844G>A (p.V282M)	Exon 7	p.[V282M];[L308Ffs*6]	PA	f	11 yrs	17-OHP: 17.1 ng/mL, DHEA-S: 121 μg/dL, Δ4-A: 1.68 ng/mL	17-OHP (ng/mL)	6.5	57.6	71.6	0.00001238	LP (PM2, PP2, PM3, PM5)	[32]
							F (μg/dL)	6.56	11.7	13.64			

PA: Premature Adrenarche, PHI: Pubic Hair of Infancy, PRA: Plasma Renin Activity, T: Testosterone, f: Female, m: Male, yrs: years, mos: months. Reference range: 17-OHP (ng/mL): 0.25–1.45 (males 6–11 months), 0.2–0.5 (females 6–9 years), 0.2–0.7 (females 10–11 years), 0.15–0.5 (males 10–11 years), 0.25–1.9 (females 12–13); Cortisol(μg/dL): 6.2–19.4; Testosterone(ng/dL): 2–7 (males 6–12 months), 2–20 (females 6–9 years), 5–25 (females 10–11 years), 10–80 (females 12–14 years); DHEA-S(μg/dL):3.4–123.6 (1–12 months), 2.8–85.2 (5–10 years), 33.9–280 (female 11–14 years), 24.4–247 (male 11–14 years); Δ4-A (ng/mL):0.05–0.3 (6–12 months), 0.05–0.55 (3–9 years), 0.1–0.8 (10–11 years), 0.15–2.5 (females 12–17 years); ACTH (pg/mL):7–64, PRA (ng/mL/h):0.5–4.7 (upright posture); Aldosterone (ng/dL): 5–132 (<1 year), 4–76 (5–8 years), 3–28 (9–12 years). The c.92C>T corresponds to p.P31L. According to the Human Genome Variation Society recommendations, the protein sequence number should not be stated in a genotype in which 1 of the 2 variants is present in a noncoding sequence. # The p.R255K has been previously reported in the literature (retrieved from dbSNP) but never reported in patients.

## Data Availability

The data presented in this study are available on request from the corresponding author due to privacy/ethical reasons.

## Appendix A

**Table A1:** Classification of pathogenic variants identified in compound heterozygotes and homozygotes presented with the classical form of 21-OHD. The genotypes identified are also presented.

Classic Form of 21-OHD	Compound Heterozygotes	Homozygotes
**SW**	**Group Null Variants in Both Alleles (2 Cases)**	**Group A Variant (3 Cases)**
	c.[1069C>T];[949C>T], c.[955C>T;1360C>T];[(?_-163)_(939 + 39_?)del]	c.[293-13C>G];[293-13C>G]
	**Group Null variant ** ***in trans*** ** with a Group A variant (2 cases)**	
	c.[1069C>T];[293-13C>G], c.[293-13C>G];[(?_-163)_(939 + 39_?)del]	
**SV**	**Group B variant ** ***in trans*** ** with a Group A variant (3 cases)**	**Group B variant (1 case)**
	c.[518T>A];[293-13C>G]	c.[518T>A];[518T>A]
	**Group B variant ** ***in trans*** ** with a null variant (3 cases)**	
	c.[518T>A];[332_339del], c.[332_339del];[1451G>C], c.[518T>A];[(?_-163)_(939 + 39_?)del]	

All variants are described herein at the DNA level. The corresponding description at the protein level is presented below. c.1069C>T=p.R357W, c.581T>A=p.I173N, c.332_339del=p.Gly111Valfs*21, c.949C>T=p.Q317* c.955C>T=p.Q319*, c.1451G>C=p.R484P c.1360C>T=p.P454S. The (?_-163)_(939 + 39_?)del corresponds to deletion of the *CYP21A2* gene based on the genomic coordinates of the MLPA probes. The c.293-13C>G corresponds to the I_2_splice site pathogenic variant.
